# Developmental Neurotoxicity of Pyrethroid Insecticides: Critical Review and Future Research Needs

**DOI:** 10.1289/ehp.7254

**Published:** 2004-10-14

**Authors:** Timothy J. Shafer, Douglas A. Meyer, Kevin M. Crofton

**Affiliations:** ^1^Neurotoxicology Division, National Health and Environmental Effects Research Laboratory, Office of Research and Development, U.S. Environmental Protection Agency, Research Triangle Park, North Carolina, USA; ^2^Curriculum in Toxicology, University of North Carolina at Chapel Hill, Chapel Hill, North Carolina, USA

**Keywords:** age-dependent toxicity, biologically based dose–response model, developmental neurotoxicity, mode of action, physiologically based pharmacokinetic model, pyrethroid, risk assessment, voltage-sensitive sodium channel

## Abstract

Pyrethroid insecticides have been used for more than 40 years and account for 25% of the worldwide insecticide market. Although their acute neurotoxicity to adults has been well characterized, information regarding the potential developmental neurotoxicity of this class of compounds is limited. There is a large age dependence to the acute toxicity of pyrethroids in which neonatal rats are at least an order of magnitude more sensitive than adults to two pyrethroids. There is no information on age-dependent toxicity for most pyrethroids. In the present review we examine the scientific data related to potential for age-dependent and developmental neurotoxicity of pyrethroids. As a basis for understanding this neurotoxicity, we discuss the heterogeneity and ontogeny of voltage-sensitive sodium channels, a primary neuronal target of pyrethroids. We also summarize 22 studies of the developmental neurotoxicity of pyrethroids and review the strengths and limitations of these studies. These studies examined numerous end points, with changes in motor activity and muscarinic acetylcholine receptor density the most common. Many of the developmental neurotoxicity studies suffer from inadequate study design, problematic statistical analyses, use of formulated products, and/or inadequate controls. These factors confound interpretation of results. To better understand the potential for developmental exposure to pyrethroids to cause neurotoxicity, additional, well-designed and well-executed developmental neurotoxicity studies are needed. These studies should employ state-of-the-science methods to promote a greater understanding of the mode of action of pyrethroids in the developing nervous system.

Pyrethroid insecticides have been used in agricultural and home formulations for more than 30 years and account for approximately one-fourth of the worldwide insecticide market (Casida and Quistad 1998). Currently, 16 pyrethroids are registered for use in the United States in a large variety of agricultural or consumer products (Bryant and Bite 2003). Often, pyrethroids are sold and/or used as mixtures containing a combination of two or more compounds (Farm Chemicals Handbook 1997). Exposure to pyrethroids has been widely documented in humans, including exposure of pregnant women, infants, and children (Berkowitz et al. 2003; Huedorf et al. 2004; Schettgen et al. 2002; Whyatt et al. 2002). Although the acute toxicity of these compounds to adults has been well characterized, the potential for developmental toxicity of pyrethroids is not well understood.

In the present review we focus on the potential for neurotoxicity after developmental exposure to pyrethroid insecticides. We also consider the current state and quality of scientific data that could be used to support risk decisions related to pyrethroid developmental and age-dependent neurotoxicity. Specifically, in this review we *a*) provide a brief overview of the toxicity of this class of compounds; *b*) review pyrethroid effects on voltage-sensitive sodium channels (VSSCs), a primary mode of action of pyrethroids; *c*) discuss the developmental profiles of VSSCs; *d*) provide examples of the results of perturbation of VSSCs during development by other insults; *e*) discuss the evidence for age-related sensitivity to this class of compounds; *f* ) summarize and critique studies of pyrethroid neurotoxicity after developmental exposure; and *g*) make recommendations regarding future research needs related to the developmental neurotoxicity of pyrethroids.

In addition to being important to scientists interested in characterizing the neurotoxicity of these compounds, this information will be useful when considering the scientific data needed to inform risk decisions related to pyrethroid insecticides. Under the Food Quality Protection Act (FQPA; 1996), the U.S. Environmental Protection Agency (EPA) is required to include a default 10× safety factor (uncertainty factor) in risk decisions to protect against potentially greater sensitivity of developing individuals to toxic insult. This factor can be adjusted only if compelling scientific data exist regarding age-related differences in sensitivity. Furthermore, developing individuals must be considered under FQPA requirements for cumulative risk assessments (classes of compounds with the same mode of action). The quality of the scientific data used to support these and other risk decisions is an important component of scientifically based risk assessment. In addition, information regarding mode of action improves the scientific basis for risk decisions (Brock et al. 2003; Mileson et al. 1998; Sonich-Mullin et al. 2001), including those related to developmental neurotoxicity (Costa 1998; Tilson 2000a, 2000b).

The U.S. EPA has recently released the revised cumulative risk assessment of the organophosphate class of insecticides (U.S. EPA 2002) and has requested that registrants of these insecticides submit developmental neurotoxicity studies to the agency. In the near future, the U.S. EPA must consider developmental and cumulative risk for other classes of insecticides, including pyrethroids. Thus, in this review we focus on issues of mode of action and age-dependent and developmental neurotoxicity as related to risk decisions under the FQPA.

## Overview of Pyrethroid Toxicity

**Figure 1:**
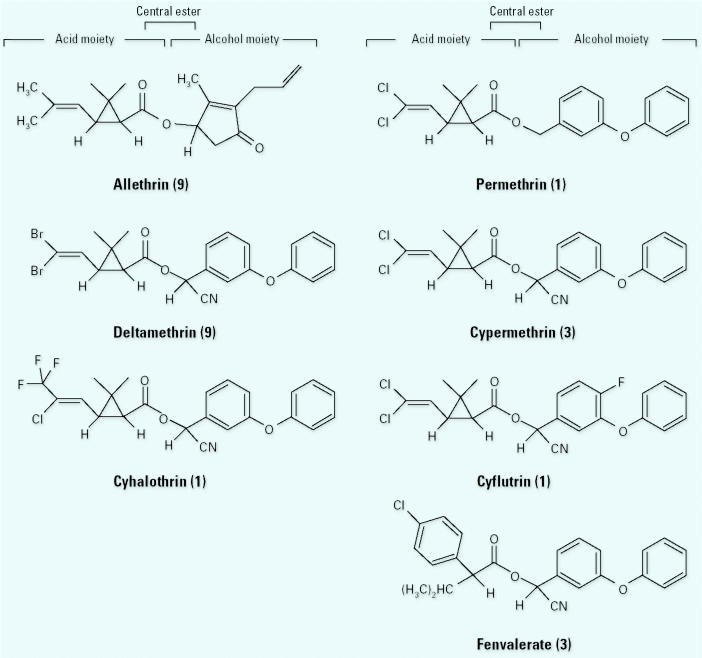
Structures of pyrethroids for which developmental neurotoxicity has been examined. Developmental neurotoxicity studies have been conducted using either technical compound or formulations of the seven pyrethroids illustrated; the numbers in parentheses after each compound name indicate the number of studies that have been conducted using that compound or a formulation containing that compound. Only one stereoisomer is illustrated for each compound.

**Figure 2:**
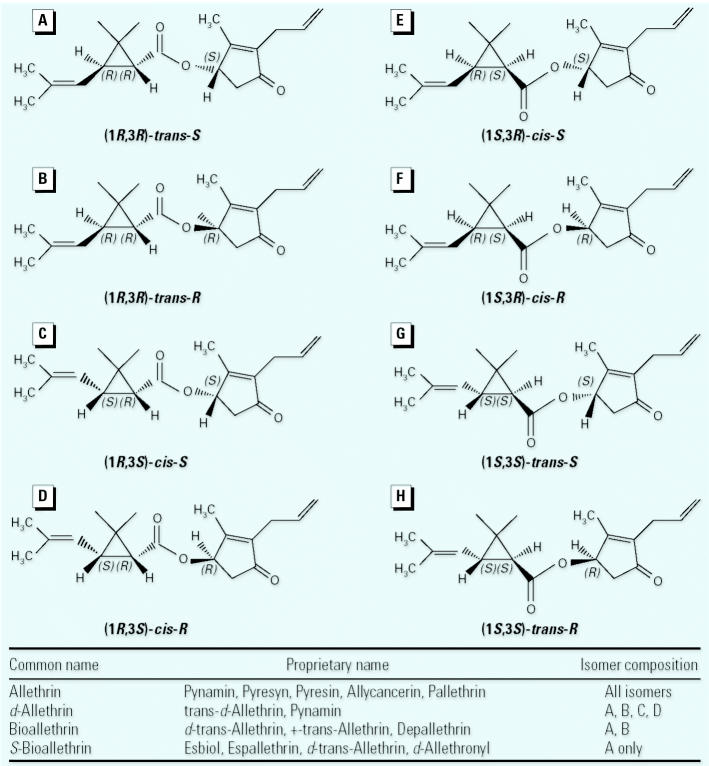
Eight possible stereoisomers of allethrin (*A*–*H*). The inset lists allethrin-containing products and the stereoisomer content of each.

The pyrethroid class of insecticides was derived from natural compounds (the pyrethrins) isolated from the *Chrysanthemum* genus of plants (Casida 1980). Although natural pyrethrins do have insecticidal activity, they also are inherently unstable when exposed to light. Therefore, the pyrethrin structure was modified to produce more stable compounds that retained the desirable insecticidal and toxicologic properties (Valentine 1990). All pyrethroids contain several common features: an acid moiety, a central ester bond, and an alcohol moiety (Figure 1). The acid moiety contains two chiral carbons; thus, pyrethroids typically exist as stereoisomeric compounds. Furthermore, some compounds also contain a chiral carbon on the alcohol moiety, which allows for three chiral carbons and a total of eight different stereoenantiomers. Pyrethroid insecticidal activity (Elliot et al. 1974), acute mammalian neurotoxicity (Gray and Soderlund 1985), and effects on VSSCs (Lund and Narahashi 1982) are stereospecific, indicating the presence of specific binding sites. For some compounds, several commercial products are available that differ in stereoisomer content. For example, allethrin is a mixture of all possible allethrin stereoisomers, *d*-allethrin contains only the 1*R* isomers, bioallethrin contains only the 1*R*-*trans* isomers, and *S*-bioallethrin is enriched in the *S* stereoisomer of the 1*R*-*trans* isomers (Figure 2).

The acute mammalian neurotoxicity of pyrethroids has been well characterized, and several comprehensive reviews of pyrethroid toxicity, metabolism, and actions are available (Kaneko and Miyamoto 2001; Narahashi 2001; Ray 2001; Soderlund et al. 2002). Verschoyle and colleagues (Verschoyle and Aldridge 1980; Verschoyle and Barnes 1972) conducted structure–activity relationship studies with a series of pyrethroids and described two generalized syndromes after acute exposure. Based on toxic signs in the rat, pyrethroids have been divided into two types: *a*) compounds that produce a syndrome consisting of aggressive sparring, increased sensitivity to external stimuli, and fine tremor progressing to whole-body tremor and prostration (type I or T syndrome); and *b*) compounds that produce a syndrome consisting of pawing and burrowing, profuse salivation, and coarse tremor progressing to choreoathetosis and clonic seizures (type II or CS syndrome) (Verschoyle and Aldridge 1980). Analogous toxic signs have been observed in mice (Lawrence and Casida 1982; Staatz et al. 1982) and cockroaches (Gammon et al. 1981; Scott and Matsumura 1983). Structurally, a key difference between type I and type II pyrethroids is the absence or presence, respectively, of a cyano group at the α carbon of the 3-phenoxybenzyl alcohol moiety of the compound. Thus, the type I/II or T/CS nomenclatures are useful as general classification schemes and are widely used in the published literature. However, a few pyrethroids do not fit neatly into these schemes because they produce signs related to both syndromes (Verschoyle and Aldridge 1980; for review see Soderlund et al. 2002). Further, these schemes are based on doses of pyrethroids that cause overt neurotoxicity and thus may not apply to either low-dose or developmental exposures. Because it conveys useful structural information, in this review we use the type I/II classification system.

## Effects of Pyrethroids on VSSCs

**Figure 3:**
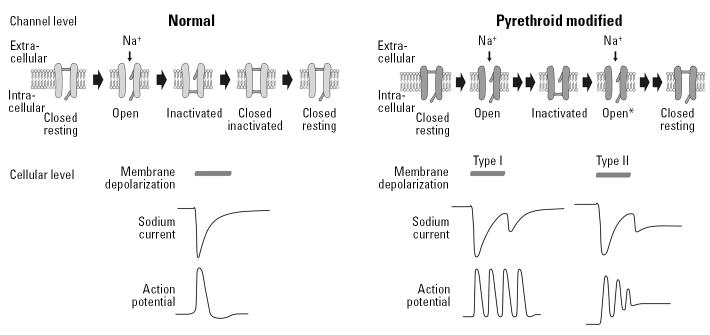
Pyrethroid effects on neuronal excitability. This schematic depicts pyrethroid effects on individual channels, whole-cell sodium currents, and action potentials. Depolarization opens VSSCs (top left) allowing sodium to enter the cell. To limit sodium entry and depolarization length, VSSCs inactivate and must return to a “resting” state before reopening. Pyrethroids inhibit the function of two different “gates” that control sodium flux through VSSCs (top right), delaying inactivation (indicated by double arrows between states) of the channel and allowing continued sodium flux (Open*). If sodium current through an entire cell is measured, depolarization leads to a rapidly inactivating current under normal circumstances (bottom left, Sodium current). Pyrethroid-modified VSSCs remain open when depolarization ends (bottom right, Sodium current), resulting in a “tail” current (the notch at the end of example currents). If membrane voltage is examined, depolarization under normal circumstances generates a single action potential (bottom left). VSSCs modified by type I compounds (bottom right, Action potential) depolarize the cell membrane above the threshold for action potential generation, resulting in a series of action potentials (repetitive firing). Type II compounds cause greater membrane depolarization, diminishing the sodium electrochemical gradient and subsequent action potential amplitude. Eventually, membrane potential becomes depolarized above the threshold for action potential generation (depolarization-dependent block).

The primary mode of pyrethroid action in both insects and mammals is disruption of VSSC function. Perturbation of sodium channel function by pyrethroids is stereospecific (Lund and Narahashi 1982); those stereoisomers that are the most potent disruptors of VSSC function also have the most potent insecticidal or toxicologic activity (Ray 2001). Pyrethroids slow the activation, or opening, of VSSCs. In addition, they slow the rate of VSSC inactivation (or closing) and shift to more hyperpolarized potentials the membrane potential at which VSSCs activate (or open) (for review, see Narahashi 1996). The result is that sodium channels open at more hyperpolarized potentials (i.e., after smaller depolarizing changes in membrane potential) and are held open longer, allowing more sodium ions to cross and depolarize the neuronal membrane. In general, type II compounds delay the inactivation of VSSCs substantially longer than do type I compounds. Type I compounds prolong channel opening only long enough to cause repetitive firing of action potentials (repetitive discharge), whereas type II compounds hold open the channels for such long periods of time that the membrane potential ultimately becomes depolarized to the point at which generation of action potentials is not possible [depolarization-dependent block (Figure 3)]. These differences in prolongation of channel open times are hypothesized to contribute to the differences in the CS and T syndromes after exposure to type II and I pyrethroids, respectively (for review, see Ray 2001).

Mammalian VSSCs are composed of one α and two β subunits. Ten separate α subunits (Table 1; Ogata and Ohishi 2002) and four different β subunits (Isom 2002) have been identified and are expressed in a tissue-, region-, and time-specific manner. With one exception (the Na_X_ subunit), α subunits all comprise VSSCs when expressed individually or with β subunits. The α subunit forms the pore of the channel and determines its major functional characteristics, whereas the β subunits are auxiliary proteins that influence gating properties, localization in the membrane, and interactions with cytoskeletal proteins (Isom 2001, 2002). The diverse functional roles of VSSC, such as generating action potential spikes, amplifying sub-threshold depolarizations, regulating repetitive firing and generating after-depolarizations, depend on the numerous potential combinations of α and β subunits (Ogata and Ohishi 2002). The types of VSSCs expressed in different regions, their relative sensitivity, and their functional role may all contribute to the manifestation of pyrethroid effects.

## VSSC Heterogeneity and Pyrethroid Effects

All available evidence indicates that pyrethroids bind to the α subunit of the VSSC. Trainer et al. (1997) expressed only the Na_v_1.2 α subunit in Chinese hamster ovary cells and found that the presence of the α subunit is sufficient for pyrethroids to produce their characteristic effects on sodium channel function in mammalian cells. This conclusion is supported by additional research demonstrating that pyrethroids alter currents produced by expression of Na_v_1.2 (Smith and Soderlund 1998) or Na_v_1.8 (Smith and Soderlund 2001) in oocytes in the absence of coexpression with β subunits. Interestingly, coexpression of the β1 subunit with Na_v_1.2 increased the sensitivity of this channel compared with expression of Na_v_1.2 alone (Smith and Soderlund 1998), indicating that the β subunit modulates the affinity of pyrethroid interaction with the channel. Mutations in the α subunit of both insects (Lee and Soderlund 2001; Smith et al. 1997) and mammals (Vais et al. 2000, 2001; Wang et al. 2001) alter the sensitivity of VSSCs to pyrethroid effects, supporting the conclusion that pyrethroids interact with the α subunit.

The relative susceptibility of the 10 different VSSC α subunits to pyrethroids is not well understood. Differential sensitivity of VSSCs to pyrethroids was first reported by Tatebayashi and Narahashi (1994). In a comparison of tetramethrin effects on tetrodotoxin-sensitive (TTX-S) versus -resistant (TTX-R) sodium channels in dorsal root ganglion neurons, TTX-R channels were demonstrated to be more sensitive to perturbation by tetramethrin (Tatebayashi and Narahashi 1994). However, TTX-R or TTX-S channels may arise from several different VSSC α subunits (Table 1). Although not all α subunits have been examined, differences in sensitivity to pyrethroids were reported after expression of different subunits *in vitro* (details provided in Table 1). For example, Na_v_1.2 (Smith and Soderlund 1998) is sensitive to type II but not type I compounds, whereas Na_v_1.8 (Smith and Soderlund 2001) is sensitive to both. Interactions of pyrethroids with other sodium channel α subunits have not been investigated to date. Importantly, the pyrethroid sensitivity of VSSC subunits and splice variants expressed during development has yet to be examined.

## Developmental Expression of VSSC

VSSCs show complex regional and temporal ontogeny, which is briefly summarized in Table 1. In general, embryonically expressed forms of VSSCs are replaced by expression of adult forms as neurodevelopment proceeds. For example, high expression of Na_v_1.3 during embryonic periods (Albrieux et al. 2004) diminishes as expression of Na_v_1.2 increases in early postnatal periods in rodents (Felts et al. 1997), and expression of Na_v_1.2 at immature nodes of Ranvier is replaced by Na_v_1.6 as myelination proceeds (Boiko et al. 2001; Jenkins and Bennett 2002). Similar changes are observed with the β subunits, because β3 expression is replaced by β1 and β2 (Shah et al. 2001). Alternatively spliced forms of the VSSC subunits also contribute to developmental differences in expression because the Na_v_1.2, Na_v_1.3, and Na_v_1.6 subunits all have splice variants that are expressed in rodents from embryonic through early postnatal ages (Gustafson et al. 1993; Plummer et al. 1997; Sarao et al. 1991). Given the previously reported differences in α subunit sensitivity to pyrethroids, the complex ontogeny of VSSC expression could result in altered sensitivity (either increases or decreases) of the developing nervous system to perturbation by various pyrethroids. In addition, understanding the timing and localization of expression of the most pyrethroid-sensitive VSSCs during neurodevelopment could help in understanding and explaining effects reported after developmental exposure. With respect to age-dependent toxicity of pyrethroids, research to date indicates that toxicokinetic and not toxicodynamic factors account for differences in susceptibility between young and adult animals (Cantalamessa 1993; Sheets et al. 1994); however, toxicodynamic factors have not been systematically examined.

## Disruption of VSSC Function and Expression during Development

Evidence from mutation and knockout models demonstrates that perturbation of VSSC function during development impairs nervous system structure and function. Several examples are discussed below for illustrative purposes. These examples demonstrate the plausibility that perturbations in VSSC function by pyrethroids during development could result in adverse consequences in the developing nervous system.

Knockout and mutant mouse models of sodium channel α subunits demonstrate varying degrees of adverse outcomes associated with loss or alteration of specific channel subunits. When mRNA for the Na_v_1.2 subunit was reduced by approximately 85%, mice exhibited reduced levels of electrical excitability, had high levels of apoptotic neurons in the brainstem and cortex, and died from severe hypoxia within 1–2 days of birth (Planells-Cases et al. 2000). In contrast, mutation of the gene encoding the Na_v_1.6 subunit resulted in development of hindlimb paralysis, skeletal muscle atrophy by postnatal day (PND)10, and death by PND20 (Porter et al. 1996). Atrophy was specific to muscle innervated by spinal and not oculomotor neurons (Porter et al. 1996). Finally, Na_v_1.8 knockout mice survived to adulthood and exhibited normal behavior, although sensation of some types of noxious stimuli was lost or diminished (Akopian et al. 1999; Laird et al. 2002).

In humans, perturbation of nervous system development has been associated with altered VSSC structure or function. Recent advances in molecular genetics have identified in genes coding for VSSC subunits a number of mutations that result in neuronal hyperexcitability due to subtle changes in channel gating and inactivation (see Meisler et al. 2001, their Table 3). These mutations have been linked to various forms of epilepsy in humans, providing evidence that changes in VSSC function can give rise to clinically definable disease (Claes et al. 2001; Escayg et al. 2001; Meisler et al. 2002; Noebels 2002; Wallace et al. 2001). Mouse models expressing these mutant ion channels have been constructed, facilitating the study of these diseases (Kearney et al. 2001; Meisler et al. 2001). It is noteworthy that pyrethroids, like these mutations, alter VSSC activation, inactivation, and neuronal excitability. The mechanisms and magnitude of mutational versus pyrethroid effects are different, as would be the duration of effect (dependent on exposure for pyrethroids vs. permanent for mutations). Because of these differences, results from mutation and knockout models may not be predictive of developmental exposure to pyrethroids. Notably, potential interactions between pyrethroids and these mutations to VSSCs have not yet been examined.

Phenytoin, an anticonvulsant that blocks VSSCs as well as other ion channels (Catterall 1999), has been demonstrated to disrupt nervous system structure and function after developmental exposure (Adams et al. 1990). In humans, the use of anticonvulsants during pregnancy has been associated with a number of defects and malformations, which collectively are referred to as fetal hydantoin syndrome, and include microcephaly and intellectual impairment. Studies in animal models support the human findings (Hatta et al. 1999; Ohmori et al. 1997, 1999; Schilling et al. 1999; Vorhees et al. 1995). Thus, developmental exposure to this drug, which acts on VSSCs, can produce significant alterations in nervous system structure and function. It should also be noted that, although phenytoin is used as an example, there are currently no data to suggest that developmental exposure to pyrethroids results in similar effects.

## Age-Related Differences in Sensitivity to Pyrethroids

The magnitude of the age-related toxicity of pyrethroids appears to be much larger than for many other pesticide classes, but the number of studies is small. Whether this age-related neurotoxicity includes both type I and type II compounds is currently unclear. In neonatal versus adult rats, the acute lethality of the type II pyrethroid deltamethrin was 16-fold greater in young animals (Sheets et al. 1994). Concentration data indicate that the age dependency was due to lower metabolic capabilities in the young rats (Sheets et al. 1994). Similarly, the type II pyrethroid cypermethrin was 17-fold, and the type I pyrethroid permethrin was 6-fold more lethal in PND8 rats compared with adults; metabolic inhibitors were used to demonstrate that toxicokinetic factors were responsible for this age-dependent susceptibility (Cantalamessa 1993). In contrast, evidence has been presented that two type I pyrethroids, cismethin and permethrin, did not have any age-dependent toxicity (Sheets 2000).

Age-related sensitivity to pyrethroids may be influenced by dose. In a symposium report, Sheets (2000) argued that the age-dependent sensitivity of pyrethroids is apparent only at high acute doses. This report contained data suggesting a lack of age-dependent differences in the behavioral toxicity of type I and type II pyrethroids at doses below those causing overt toxicity. However, age-dependent differences in pyrethroid neurotoxicity have not been thoroughly studied at the lower end of the dose–response relationship (sublethal doses). The scientific basis for decisions related to the FQPA could be strengthened by additional studies comparing the relative susceptibility of differential sensitivity between young and adult animals, particularly at sublethal doses. For example, replication of Sheets’s (2000) report and expansion to include additional compounds would provide useful information regarding sensitivity differences between developing and adult animals.

## Pyrethroid Developmental Neurotoxicity Studies

A total of 22 studies were evaluated for this review (Tables 2–4), including 19 peer-reviewed publications (Table 2), unpublished studies (Muhammad and Ray, unpublished data; see Table 3), and regulatory studies provided by Bayer AG (Table 4; Ivens et al., unpublished data; Jekat et al., unpublished data). The studies conducted by Muhammad and Ray (unpublished data) consisted of several similarly treated “cohorts” for both *S*-bioallethrin and deltamethrin. Rather than present the overall findings for each of these two compounds, the results of individual “cohorts” are summarized in Table 3 to provide more detailed information. Tables 2–4 contain a summary of important information from each study, including test compound/formulation, animal species, dosing period, and major findings. Because the vehicle used and route of exposure can have profound influence on the expression of pyrethroid neurotoxicity in adult rats (Crofton et al. 1995), this information is included as well.

Allethrin (in the form of allethrin, *d*-allethrin, bioallethrin, and *S*-bioallethrin) and permethrin are the only type I pyrethroids for which peer-reviewed studies of potential developmental neurotoxicity have been conducted. Of the type II compounds, results of developmental studies have been published for deltamethrin, cypermethrin, fenvalerate, and cyhalothrin, and data regarding the developmental neurotoxicity of cyfluthrin (Jekat et al., unpublished data) have been submitted to the U.S. EPA. Thus, no developmental neurotoxicity studies exist for many pyrethroids.

Rodents were the sole animal models used in these studies: 13 studies used rats and 9 studies used mice. No studies were conducted specifically to examine species differences, nor could any clear species-dependent effects be discerned. The choice of rats or mice seemed to be based on *a*) previous use of that species in the laboratory or *b*) whether or not the study was designed to replicate (in whole or part) results published previously by other investigators. A systematic comparison of factors that underlie potential species differences in neurotoxic responses could provide useful information regarding the extrapolation of data from animals to humans. For example, Na_v_1.3 expression in rodents appears to be primarily embryonic, yet in humans considerable expression in adults has been reported (Whitaker et al. 2000, 2001). How this and other species differences influence neurotoxic responses has not been investigated.

Several studies reported persistent changes in behavior and/or neurochemistry in animals examined long after exposure had stopped. Eriksson’s group (Ahlbom et al. 1994; Eriksson and Fredriksson 1991; Eriksson et al. 1993; Eriksson and Nordberg 1990) has reported that mice exposed to pyrethroids during PND10–16 exhibit increased motor activity and lack of habituation, as well as changes in density of muscarinic acetylcholine receptor (mAChR) binding for as long as 5 months (Talts et al. 1998) after cessation of exposure. Given the short half-lives for pyrethroids (Anadón et al. 1991, 1996; for review, see Kaneko and Miyamoto 2001), these effects are likely due to exposure during development and not residual tissue concentrations of pyrethroids. Studies conducted by Eriksson and co-workers used bioallethrin and deltamethrin, which contain only two and predominantly one stereoisomer, respectively. Thus, effects can be ascribed to the compound that has insecticidal activity (vs. studies conducted with formulated products). In addition, dose–response relationships have been demonstrated for bioallethrin (Ahlbom et al. 1994), and the replication of effects, both behavioral and biochemical, within this laboratory has been consistent over several studies. Others have also reported persistent changes in behavior and/or biochemistry, including learning (Moniz et al. 1990), motor activity (deltamethrin only; Husain et al. 1992), sexual behavior (Lazarini et al. 2001), mAChR binding (Aziz et al. 2001; Malaviya et al. 1993), and blood–brain barrier permeability (Gupta et al. 1999a).

There were several studies that examined both motor activity and mAChR expression after developmental exposure to pyrethroids. A summary of effects on these end points, independent of dose, exposure period, and other parameters, is provided in Table 5. In all of these studies, quinuclidinyl benzilate (QNB) binding was used to measure mAChR expression. QNB is a nonspecific antagonist for this receptor (Watling et al. 1995) and does not discriminate between mAChR subtypes (M1–M5). Measurement of QNB binding may in fact be one of the more comparable end points across these numerous studies. In addition, many but not all of these studies examined mAChR expression at PND17 and/or 4 months of age.

Comparison of pyrethroid effects on QNB binding across studies does not reveal clear trends in reported effects between laboratories. In preweanling animals, across all compounds and treatment protocols, QNB binding was reported to increase in six studies, decrease in two studies, and not change in four studies (Table 5). In cortical tissue, the data for PND17 are more consistent in that five of eight studies reported increases in mAChR expression. If only the various forms of allethrin are considered, four studies reported increases and two reported no change in QNB binding when measured on PND17. Persistent alterations in mAChR in adulthood after developmental exposure are less clear, with three studies reporting increases, three reporting decreases, and five reporting no change in QNB binding. Considering only allethrin forms again, QNB binding increased or decreased in two studies each and was unchanged in three studies.

Differences in a number of important variables may underlie some of the inconsistencies in QNB binding data. One difference is exposure route. Two studies used inhalation exposure (Ivens et al., unpublished data; Jekat et al., unpublished data), whereas exposure in the remainder of the studies was via oral gavage (Table 5). A comparison of effects in Tables 2–5 suggests that this is not a tenable explanation for these inconsistencies because results do not correlate to route. Another variable that differed between laboratories was the formulation of allethrin used. Allethrin, like all pyrethroids, exists as several different stereoisomers (Figure 2), and the insecticidal and toxic effects of pyrethroids are highly stereospecific. These studies employed allethrin formulations with differing contents of allethrin stereoisomers; two groups used *d*-allethrin (Ivens et al., unpublished data; Tsuji et al. 2002), one used bioallethrin (Eriksson group: Ahlbom et al. 1994; Ericksson and Fredriksson 1991; Eriksson and Nordberg 1990; Talts et al. 1998), and two used *S-*bioallethrin (Muhammad and Ray, unpublished data; Pauluhn and Schmuck 2003). Again, data in Table 5 suggest that this is not a tenable explanation because *d*-allethrin and bioallethrin result in either increases or no effects on mAChR binding. An additional variable in these data sets is the specific methods used in the competitive binding experiments. Competition experiments with carbachol were used in several studies to distinguish between high- and low-affinity QNB binding sites (Ahlbom et al. 1994; Eriksson and Fredriksson 1991; Eriksson and Nordberg 1990; Ivens et al., unpublished data; Jekat et al., unpublished data; Talts et al. 1998). Two studies (Ahlbom et al. 1994; Eriksson and Nordberg 1990) reported that bioallethrin increased the percentage of low-affinity binding sites in PND17 mice, an effect not reported in adult mice, despite changes in the density of muscarinic binding (Eriksson and Fredriksson 1991; Talts et al. 1998). Ivens et al. (unpublished data) did not find changes in the percentages of high- and low-affinity sites, even though they did report changes in the density of QNB binding sites in PND17 animals. In some cases, the relative proportion of high- and low-affinity sites was not investigated even though changes in density were reported (Muhammad and Ray, unpublished data). The ability to distinguish high- and low-affinity sites, and effects thereon, is dependent on the number of points included on the agonist competition curve. Studies conducted by the group at Bayer (Ivens et al., unpublished data; Jekat et al., unpublished data) used seven different concentrations of carbachol, whereas studies conducted by Eriksson’s group (Ahlbom et al. 1994; Eriksson and Fredriksson 1991; Eriksson and Nordberg 1990) used 18 concentrations of carbachol (Eriksson P, personal communication). This information was typically not available to evaluate and may account for some reported differences, because use of too few points may preclude detection of changes in the low-affinity site. Overall, the data across laboratories indicate that changes in QNB binding may not be a robust response to developmental exposure to pyrethroids and that conditions may need to be more carefully controlled in order to observe changes.

A smaller number of studies examined potential alterations in catecholaminergic systems. Both deltamethrin (Lazarini et al. 2001) and bioallethrin (Muhammad and Ray, unpublished data) were reported to increase 3,4-dihydroxyphenylacetic acid (DOPAC) levels in the adult striatum after developmental exposure. However, developmental exposure to a commercial product containing fenvalerate had no effect on monoamine levels in the striatum (Moniz et al. 1999). Malaviya et al. (1993) reported that binding of ^3^H-spiroperidol to striatal membranes from PND21 rats was decreased and increased, respectively, after gestational and lactational exposure to a commercial product containing fenvalerate, whereas binding was increased after only lactational exposure to a commercial product containing cypermethrin. Thus, similar to the muscarinic cholinergic system, the dopaminergic system may be affected by developmental exposure to pyrethroids, but studies examining this system have reported inconsistent results to date.

Eriksson and co-workers have consistently reported increased motor activity and a lack of habituation after exposure to pyrethroids (Ahlbom et al. 1994; Eriksson et al. 1993; Talts et al. 1998). A comparison of effects of pyrethroids on motor function between laboratories is not as consistent. Muhammad and Ray (unpublished data) observed effects on motor activity in some cohorts but not in others. After inhalation exposure to bioallethrin (Tsuji et al. 2002) or *d*-allethrin (Ivens et al., unpublished data), no effects on activity or habituation were reported. By contrast, inhalation exposure to cyfluthrin resulted in hyperactivity and decreased habituation in female mice (Jekat et al., unpublished data). Several additional studies also examined other measures of open field or motor activity (Gomes et al. 1991a; Husain et al. 1992, 1994; Lazarini et al. 2001). Reports of effects in these studies were also variable (Table 2). The reasons for the discrepant nature of these findings are unknown.

A small number of studies tested cognitive functions (Table 2). Two studies reported that bioallethrin exposure during PND10–16 (via different routes) had no significant effect on performance in the Morris water maze at 5 (Talts et al. 1998) and 11 (Tsuji et al. 2002) months of age. Other studies reported decreases in avoidance and Y-maze learning (Aziz et al. 2001; Husain et al. 1994; Moniz et al. 1990) or no change in avoidance behavior (Gomes et al. 1991a). A major confounder in the Y-maze and avoidance studies is the use of commercial formulations rather than technical compound.

There are several common weaknesses in the developmental studies that temper the scientific strength of some individual reports, as well as the data set when taken as a whole. A key weakness is problematic statistical analyses. Most behavioral studies [with the exception of Ivens et al. (unpublished data), Jekat et al. (unpublished data), and Tsuji et al. (2002)] used multiple pups from the same litter without correction in the statistical analysis. The sampling of multiple pups from the same litter inflates the sample size and increases the probability of a type I statistical error (Abbey and Howard 1973; Holson and Pearce 1992; Muller et al. 1985; Reily and Meyer 1984). When biochemical end points were examined, statistical analyses often lacked robustness or, in some cases, were absent. In several studies examining receptor binding, results were compared (and significant differences found) using multiple Student’s *t*-tests. Use of multiple *t*-tests can easily increase the probability of a type I error (Muller et al. 1985). These study designs should use statistical models that control for multiple comparisons (e.g., analysis of variance with appropriate post hoc test for comparisons of different group means). Meta-analyses or other statistical approaches to examine related data sets from the same and different laboratories could help strengthen conclusions when effect magnitude is small but have not been conducted to date.

An additional limitation common to these reports was a lack of tissue concentration data. None of the studies reported pyrethroid blood or brain concentrations from dams or pups. Such information would have greatly facilitated comparisons between studies and would also be useful to compare target tissue concentrations in the test species with exposure estimates in pregnant women (see Whyatt et al. 2002).

Lack of information about the stereoisomer composition and/or purity of the test compound was a serious confound in some reports. Such information is important to be able to compare studies generated in different laboratories, as discussed above for the different allethrin products. In addition, several studies used formulated products rather than purified compound (Aziz et al. 2001; Gupta et al. 1999a, 1999b; Husain et al. 1992, 1994; Malaviya et al. 1993). Formulated pesticide products typically contain solvents, emulsifying agents, petroleum distillates, and other “inerts” (Farm Chemicals Handbook 1997), many of which are known or suspected to have neurotoxic properties. Although use of formulated products may provide a more real-life exposure situation, lack of information on the content of proprietary formulations hampers comparisons between studies and often precludes attributing effects directly to the pyrethroid.

Several other limitations should also be noted. The number of time points examined in these studies typically was three or fewer, one of which was often a measurement in adult animals. Considerable ontogeny of both behavioral responses as well as biochemical end points is well established. Thus, the tendency of most studies to examine a “snapshot in time” may miss important ontogenic shifts induced by these compounds. Dosing duration and age at exposure are two other important factors. Although a number of studies examined the period of PND10–15, the choice of dosing periods in the present studies was variable, and, to date, there has not been a systematic evaluation of potentially sensitive developmental periods. An additional consideration regarding dosing periods is the differential rates of neurodevelopment in rodents versus humans. Thus, studies such as those conducted by Whyatt et al. (2002) could potentially provide important information about exposure to the developing fetus. In addition, the effects of sex were not always considered in the present studies, with a few exceptions (e.g., Gomes et al. 1991b; Moniz et al. 1999). Also related to this topic is the relative distribution of males and females in a litter. In some cases, culling information was readily available; however, many studies provided no or insufficient information to evaluate this variable.

Although not necessarily a limitation, there is a significant conceptual gap between the variety of behavioral, biochemical, and physiologic end points studied to date (Tables 1–4). The relationships, if any, between these biochemical and behavioral changes have yet to be established. In addition, the relationship between the end points examined in the present studies and the major action of pyrethroids, disruption of VSSC function, is also unknown. Only one study to date has examined changes in VSSC expression (Muhammad and Ray, unpublished data). The relationship between biochemical alterations and pyrethroid-induced developmental neurotoxicity could be strengthened by better characterization of neurochemical mode(s) of action of pyrethroid neurotoxicity. Establishing mode-of-action pathways increases confidence that reported effects are the result of pyrethroid action, particularly when the magnitude of those effects is small.

## Conclusions and Recommendations for Future Research

Several research needs in the area of developmental neurotoxicity are apparent from this review. These include additional information regarding potential differences underlying age-dependent sensitivity to pyrethroids, clarification of changes in behavioral and biochemical end points, and linking these end points to VSSCs or other cellular targets. In considering these potential areas for future research, determining the priority of addressing different research questions often depends on individual perspectives. In this context, a different conceptual approach to conducting future research may improve the resulting data’s usefulness for the purpose of risk decisions.

**Figure 4:**
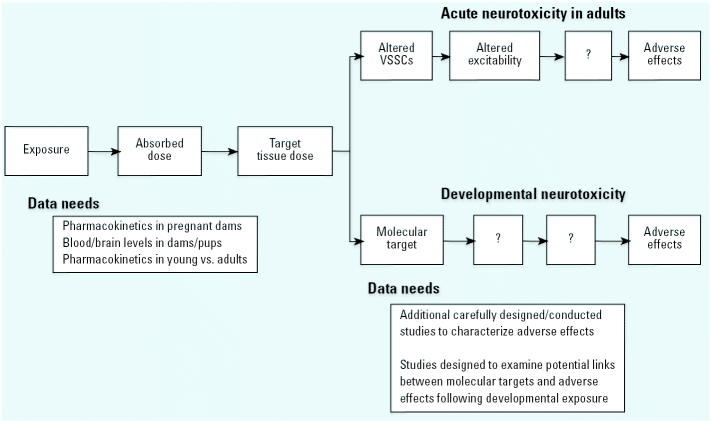
Major elements in a proposed BBDR model for pyrethroid neurotoxicity and research needs for the PBPK and PBPD components. Boxes with question marks indicate that the sequence of events between changes in the target and adverse effects has not been completely elucidated.

Biologically based dose–response (BBDR) models (Andersen and Dennison 2001) describe the relationships between different components of the continuum between exposure to and the adverse effects of a chemical (Figure 4). For example, such a model has recently been constructed for the developmental neurotoxicity of perchlorate (Jarabek et al. 2002). Mode-of-action models strengthen science in two important ways. First, the uncertainty regarding animal-to-human extrapolations can be reduced if a toxicant’s mode of action in an animal model is demonstrated to be relevant to humans (Cohen et al. 2004; Meek et al. 2003; Sonich-Mullin et al. 2001). Second, these models often provide insight into research needs by identifying data gaps and research needs. For pyrethroids, much of the future research needs can be described in the context of the type of data that would be useful in constructing a BBDR for this class of compounds, or for individual compounds within this class. A cornerstone of a BBDR model is a physiologically based pharmacokinetic (PBPK) model that describes the relationship between exposure and target tissue dose (Andersen and Dennison 2001). Additional pharmacokinetic information in animal models as well as additional pharmacokinetic and exposure information in humans is needed. For pyrethroids, this will involve defining the relationship between maternal and fetal compartments, and the involvement of oral (including lactation), inhalation, and dermal exposures to the newborn. Current data indicate that some exposure does occur to pregnant mothers, infants, and children, resulting in low internal doses (Berkowitz et al. 2003; Heudorf et al. 2004; Schettgen et al. 2002). However, insufficient information is available to adequately evaluate the range of internal doses of pyrethroids in humans. These data will be valuable in quantitative extrapolations of exposure from animals to humans (Andersen and Dennison 2001). Pharmacokinetic information is available comparing acute high-dose exposures in neonatal versus adult animals (Cantalamessa 1993; Sheets et al. 1994). However, only a limited number of compounds have been examined to date, and no information is available for ages before PND11.

Another component of a BBDR model is a physiologically based pharmacodynamic (PBPD) model (Andersen et al. 1992; Conolly 2002). PBPD models are quantitative models that describe the mode of action of a chemical. A benefit of PBPD models is identification of research gaps that are critical to link key events in the mode of action to adverse outcomes. Currently available studies of pyrethroid developmental neurotoxicity have examined a wide variety of end points but have not sought to link target tissue events (e.g., receptor activation, changes in ion channel function) to consequent biochemical, physiologic, or behavioral outcomes. Future studies need to target the large data gap between the target site (e.g., VSSCs) and adverse outcomes. For example, can the sequence of biochemical processes be described that, when perturbed by pyrethroids, result in changes in end points such as motor activity or mAChR binding? If changes in sodium currents alter neuronal firing rate, how does this then lead to alterations in neurodevelopment? Considerable information supports involvement of VSSCs in the mode of action of acute pyrethroid neurotoxicity, yet the potential role of VSSCs in developmental neurotoxicity of pyrethroids has not been examined. Future research on the developmental neurotoxicity of pyrethroids should endeavor to fill these research gaps. These studies must be designed and conducted so as to avoid the limitations mentioned in the preceding section. Such studies of the developmental neurotoxicity of these compounds can strengthen the scientific basis for risk decisions. The most efficient use of scientific resources will be to design those additional studies to fit into a BBDR scheme.

## Figures and Tables

**Table 1:** Sodium channel α subunit nomenclature and effects of pyrethroids.*^a^*

α subunit	Older names	TTX sensitivity	Tissue expression	Developmental expression	Effect of pyrethroids
Na_v_1.1	Rat I, HBSCI, GPBI, SCN1A	TTX-S	CNS, PNS, Purkinje, HP pyramidal cells, spinal motor neurons, somatic localization	Not detected in HP during development, detectable in CB Purkinje cells at PND15, detected at PND2 in SC; strong expression in motor neurons*^b^*	Not tested to date
Na_v_1.2	Rat II, HBSCII, HBA	TTX-S	CNS, forebrain, substantia nigra, HP mossy fibers, CB molecular layer, axonal localization	In HP, increase between GD17 and PND30; in CB granule cells on PND15 and Purkinje cells on PND2; detected at all ages in SC*^b^* Splice variant expressed during development*^c^*	Cypermethrin-induced tail currents detectable at > 30 nM in rat 1.2 (adult splice variant) co-expressed with β_1_ subunits; reported insensitive to permethrin or cismethrin*^d^*
Na_v_1.3	Rat III	TTX-S	CNS and DRG	HP expression at GD17, increasing at PND2, then decreasing to barely detectable at PND30. Detected at GD17 in CB neuroepithelium, decreasing thereafter, similar in SC*^b^*; developmentally regulated splice variant*^e^*	Not tested to date
Na_v_1.4	SkM1, μ1	TTX-S	Skeletal muscle	Increases with age*^f^*	Only slightly modified by 10 μM deltamethrin when expressed in HEK 293t cells*^g^*
Na_v_1.5	SkM2, H1	TTX-R	Uninnervated skeletal muscle, heart, brain	mRNA expressed in rat PND0 limbic structures and medulla; expressed in fetal and adult human brain*^h^*	Not tested to date
Na_v_1.6	NaCh6, PN4, Scn8a, CerIII	TTX-S	CNS, DRG (all diameter neurons), node of Ranvier–peripheral nerve	Truncated form expressed from GD12 to PND7, full-length mRNA expression is slight at GD14 and increases with age*^i^*	Not tested to date
Na_v_1.7	NaS, hNE-NA, PN1	TTX-S	DRG (all diameter neurons) CNS, Schwann cells	All DRG neurons at PND2, increased during development*^b^*	Not tested to date
Na_v_1.8	SNS, PN3, NaNG	TTX-R	DRG (small diameter neurons)	Expression beginning at GD15 with adult levels by PND7; largely in unmyelinated C-fibers*^j^*	Sensitive to both cismethrin and cypermethrin at thresholds of 500 nM and 30 nM, respectively *^k^*
Na_v_1.9	NaN, SNS2, PN5, NaT, SCN12A	TTX-R	DRG (small diameter neurons)	Expression beginning at GD17 with adult levels by PND7; largely in unmyelinated C-fibers *^j^*	Not tested to date
Na_x_	Na_v_2.1, Na_v_2.3 Na-G, SCL11	?	Heart, uterus, skeletal muscle, astrocytes, DRG	Transient between PND2 and 15 in HP; peak expression at PND2 in CB, SC; large DRG neurons, GD17 to PND30*^b^*	Not tested to date

Abbreviations: CB, cerebellum; CNS, central nervous system; DRG, dorsal root ganglion; GD, gestation day; HP, hippocampus; PND, postnatal day; PNS, peripheral nervous system; SC, spinal cord; TTX, tetrodotoxin; TTX-R, TTX resistant; TTX-S, sensitive to TTX.

a Data in the first four columns are based on information presented by Goldin et al. (2000) and Novakovic et al. (2001).

b Felts et al. (1997).

c Sarao et al. (1991).

d Smith and Soderlund (1998).

e Gustafson et al. (1993).

f Kallen et al. (1990).

g Wang et al. (2001).

h Donahue et al. (2000).

i Plummer et al. (1997).

j Benn et al. (2001).

k Smith and Soderlund (2001).

**Table 3:** Summary of developmental neurotoxicity studies with pyrethroid compounds in NMRI mice dosed once daily on PND10–16 (Muhammad and Ray, unpublished data).

Compound	Dose/route/vehicle	Effects	Comments
*d*-Allethrin, 93% purity (*cis/trans*) Experiment 13	0.7 mg/kg egg lecithin/peanut oil (1:10) 40% fat emulsion	4 months: 0 effect on motor activity; 0 effect on mAChR (QNB) binding in CTX	Strengths: each chemical was examined in several cohorts in this study; closely replicates methodology of Eriksson and co-workers (see Table 2) for motor activity measurements; examined vehicle differences; technical compounds of known purity (100% for deltamethrin and 95.2% for *S*-bioallethrin)
*S-*Bioallethrin (*trans*) Experiment 17a	0.7, 3.5 mg/kg egg lecithin/peanut oil (1:10) 40% fat emulsion	4 months: ↑motor activity, habituation (slow mobile counts), 0 effect on mAChR in CTX	Limitations: not published, peer-reviewed or submitted to any regulatory agency; litter was not used as statistical unit; statistical models not well described; *t*-tests used for biochemical measures; date of study unknown, circa mid-1990s
*S-*Bioallethrin (*trans*) Experiment 19a	Attempt to replicate experiment 17a	4 months: ↑mAChR in CTX, CB (3.5 mg/kg); ↑mAChR brainstem (0.7 and 3.5 mg/kg); ↓habituation (slow mobile counts) by 0.7 mg/kg dose; ↑DOPAC, HVA in striatum; ↑saxitoxin binding in CB and MB, ↓in CTX	
*S-*Bioallethrin (*trans*) Experiment 25a	0.7 mg/kg, corn oil	PND17: 0 effect on mAChR in CTX 4 months: no data provided, despite mention that motor activity and mAChR were assessed	
*S-*Bioallethrin (*trans*) Experiment 26a	0.7 mg/kg, corn oil	4 months: significant delay in habituation of slow rearing, fast rearing, total rearing, and rearing time; 0 effect on mobile activity and time, 0 effect on mAChR	
Deltamethrin Experiment 12	0.7 mg/kg, egg lecithin/peanut oil (1:10) 40% fat emulsion	4 months: ↑ rearing time fast and total mobile counts slow, fast, and total rearing; delayed habituation of counts, slow mobile counts, and mobile time mAChR not examined	
Deltamethrin Experiment 23	0.7 mg/kg, corn oil	4 months: ↑mAChR in CTX; no effect on any measure of motor activity	
Deltamethrin Experiment 25	0.7 mg/kg, corn oil	PND17: ↑mAChR; motor activity not examined	
Deltamethrin Experiment 26	0.7 mg/kg, corn oil	4 months: significant delay in habituation of slow mobile counts, mobile and rearing time; 0 change in mAChR (increased but not significant)	

Abbreviations: CB, cerebellum; CTX, cortex; HVA, homovanillic acid; MB, midbrain.

**Table 2:** Summary of peer-reviewed developmental neurotoxicity studies with pyrethroids.*^a^*

Species/compound	Dose/route/vehicle	Dosing period	Effects	Reference	Comments
Rat (Wistar)					
Cyhalothrin (type II)	0.02% in drinking water; 0.4% sucrose + “cyhalothrin vehicle”	PND0–21	↓learning avoidance latencies at PND90, 0 effect on motor activity in pup	Moniz et al. 1990	Strengths: maternal behavior examined in Moniz et al., 1990 (no effect); culling described but not even across studies (culled to 5, 6, and 8 pups/dam) Limitations: commercial product, unknown vehicle (“cyhalothrin vehicle”) composition; dosing time frame not clear, but thought to be GD0–PND0 (Gomes et al. 1991a, 1991b); inappropriate statistical models; minimal description of results; not clear that litter is statistical unit (numbers of replicates in figure legends do not always agree with number of treatment groups)
	0.018%; 1 mL dermal, daily; “cyhalothrin vehicle”	“Entire pregnancy”	Delayed development of fur, ear/eye opening, and testes descent. PND90: ↓hole-board head dips; 0 effect avoidance; and locomotion in open field	Gomes et al. 1991a	
			0 change in sexual behaviors in males or females	Gomes et al. 1991b	
Fenvalerate (type II)	10 mg/kg, i.p.; saline	GD18 and PND2–5	0 effect: testis descent, weight, monoamine levels, stereotyped behavior, locomotion, rearing ↓pup weight on PND21, ↓ductus deferens and seminal vesicle weight; female sexual behavior at PND120	Moniz et al. 1999	Strengths: litter as statistical unit; more complete and appropriate statistical analysis, but still some incorrect uses of *t*-test (Moniz et al. 1999); maternal weight examined/reported; Lazarini et al. (2001) considered sex differences; only papers examining reproductive behavior; culling, male/female ratios described and even. Housing as adults described
Deltamethrin (type II)	0.08 mg/kg, p.o. “deltamethrin vehicle”	GD6–15, once daily	PND21: ↑rearing in males; 0 effect on locomotion frequency in males or females	Lazarini et al. 2001	
			PND60 males: ↓immobility time in forced swim test ↑DOPAC, DOPAC/DA, NA 0 effect on 5HT, 5HIAA, HVA/DA; 0 effect in PND60 females		Limitations: deltamethrin commercial product; unknown (“deltamethrin vehicle”) vehicle composition; purity of fenvalerate not known; discrepancies between text and figures in Moniz et al. (1999 their Figure 3); differences in control testes descent day in Moniz et al. (1999) vs. Gomes et al. (1991, 1991b) (19 vs. 23 days)
Mouse (NMRI)					
Bioallethrin (type I)	0.72 and 72 mg/kg 20% fat emulsion (egg lecithin/peanut oil)	PND10–16, once daily	PND17: ↑mAChR density and altered ratio of high- and low- affinity QNB binding sites in CTX but not HP with deltamethrin and bioallethrin at low (0.7 mg/kg) but not high doses	Eriksson and Nordberg 1990	Strengths: consistent demonstration of increased motor activity and lack of habituation with bioallethrin and deltamethrin; dosing occurs over a critical period of brain development; dose response demonstrated for bioallethrin for behavior and biochemistry effects present 3.5–4 months postdosing; behavior, biochemistry measured in same animals; changes in mAChR binding in CTX ~10% at 4 months, but changes not observed after 5 months (bioallethrin); consistent effects over several different studies; history of publications with motor activity and QNB binding
Deltamethrin (type II)	0.71 and 1.2 mg/kg 20% fat emulsion (egg lecithin/peanut oil)		0 change in nAChR density		
Bioallethrin (type I)	0.7 mg/kg, p.o.; 20% fat emulsion (egg lecithin/peanut oil)		4 months: ↑motor activity with lack of habituation; ↓mAChR density in CTX; 0 change in mAChR in HP, STR	Eriksson and Fredriksson 1991	
Deltamethrin (type II)	0.7 mg/kg, p.o.; 20% fat emulsion (egg lecithin/peanut oil)		4 months: ↑motor activity with lack of habituation; 0 change in mAChR in CTX, HP, STR		Limitations: statistical analysis of biochemical data increases the possibility of type I error; unclear that litter is unit of treatment; in some cases, changes as small as 1–3% reported as significant (biochemistry); sex differences not considered/included; toxicity observed at high dose of deltamethin and bioallethrin by Eriksson and Nordberg (1990), with tolerance developing by the fourth day of dosing
Bioallethrin	0.42, 0.70, 42 mg/kg, p.o.; 20% fat emulsion (egg lecithin/peanut oil)		PND17: ↑mAChR density in CTX; ↑low-affinity QNB (mAChR) binding 4 months: ↑motor activity with lack of habituation; ↓mAChR density in CTX	Ahlbom et al. 1994	
Bioallethrin	0.7 mg/kg, p.o.; 20% fat emulsion (egg lecithin/peanut oil) 4 treatment groups: vehicle as pup and 5 months; VB, vehicle as pup, bioallethrin at 5 months; BV, bioallethrin as pup, vehicle at 5 months; BB, bioallethrin as pup and 5 months	PND10–16, once daily; again at 5 months for 7 days, once daily	5 months: ↓motor activity with lack of habituation in BB and BV groups Performance in H_2_O maze: reversal in BB groups; 0 effect in BV, VB groups mAChR density in CTX: ↑in BB treatment group; 0 effect in BV, VB groups	Talts et al. 1998	
Rat (Wistar)					
Deltamethrin (type II)	0.7 mg/kg, i.p.; propylene glycol	PND9–13	Examined on PNDs 12, 15, 21, and 30: delayed cerebellar cytogenesis and morphogenesis of interneurons, vascular damage with focal degeneration; ↓brain and body weight	Patro et al. 1997	Strengths: only study examining morphology; culled litters to equal numbers; time course examined; within- litter dosing design Limitations: effects may be due to decreased growth, not direct neurotoxicity; inappropriate statistical models; toxicity; inappropriate statistical models; no control for “maternal” neglect effects in control vs. treated pups
Rat (Druckrey)					
Cypermethrin	Experiment 2: 5 mg/kg, p.o. (corn oil vehicle)	PND10–13, 17, or 30	↑BBB permeability at PNDs 13, 17, and 30 by 71, 61, and 80%; effect recovered by PND60 following withdrawal on PND18	Gupta et al. 1999a	Strengths: control data demonstrate maturation of BBB; within-paper replication of effect; technical grade (94.5% purity) cypermethrin*^b^* Limitation: litter was not the statistical unit
	Experiment 3: 2.5 mg/kg, p.o. (corn oil vehicle), (1/100 LD_50_)	PND10–17	↑BBB permeability by 28%		
Allethrin	18 hr/day inhalation of vapors; unknown commercial product containing 3.6% Allethrin, 96% kerosine, 0.3% stabilizer	PND2–19	↓body (23%) and brain (17%) weights; ↑BBB permeability, LH levels on PND10 but not PND18; ↑(small) in conjugated dienes (measure of lipid peroxidation) on PND10; ↓GSH 17% on PND10; ↑GSH by 28% on PND18	Gupta et al. 1999b	Strengths: replication of fluorescence levels on PND10 compared with Gupta et al. (1999a); litters culled to 8 pups/dam (size of litter is known) Limitations: unknown formulation; exposure to kerosine > > allethrin; no kerosine control
Deltamethrin	1.0 mg/kg, p.o., deltamethrin formulation in corn oil	GD14–20	Delayed surface righting reflex 6 and 12 weeks postnatal: ↑AChE activity; ↑GAP-43 immunohistochemistry (both % area and total number of positive cells); ↓QNB *B*_max_; ↓relearning in Y-maze task	Aziz et al. 2001	Strengths: examined two time points; behavioral and biochemical changes Limitations: unknown formulation, corn oil used as “control”; unclear that litter is statistical unit; maze learning procedure is poorly described, and “relearning” is poorly defined
Rat (Wistar)					
Deltamethrin	7 mg/kg, p.o. 2.8% EC formulation, peanut oil	GD5–21	↓weight of unspecified brain regions at PND22(?);↑resorptions and neonatal death; delayed surface righting, eye opening, fur development, incisor eruption, and pinna detachment; ↓grip strength; ↓motor activity at PNDs 21 and 42; altered regional polyamine levels	Husain et al. 1992	Strengths: work uniquely covers effects of pyrethroids on different periods of perinatal development from shortly after conception to postweaning, and suggests that effects may depend on the exposure period (includes Malaviya et al. 1993). However, different compounds were utilized; effects on maternal parameters, general toxicity recorded; litter size adjusted to an average of 8 pups/litter
Fenvalerate	10 mg/kg, p.o.; 20% EC formulation, peanut oil		Delayed surface righting, eye opening, fur development, incisor eruption, and pinna detachment; ↓grip strength; 0 effect on motor activity; altered regional polyamine levels		Limitations: formulated products used; lack of relevant vehicle controls; general or less specific toxicity may be indicated by changes in fur development, pinna detachment; statistical models are often inappropriate; descriptions of comparisons (data sets) used for statistical tests are sometimes unclear or confusing; not clear that litter is the statistical unit
Cypermethrin	15 mg/kg, p.o.; 25% EC formulation; peanut oil		Delayed surface righting, eye opening, fur development, incisor, eruption and pinna detachment; 0 effect on motor activity; altered regional polyamine levels		
Deltamethrin	7 mg/kg; 2.8% EC formulation, corn oil	PND22–37	↓hippocampal weight without effect on other brain regions; ↑mitochondrial monamine oxidase and microsomal AChE without effect on Na/K ATPase; ↑spontaneous locomotor activity; ↓conditioned avoidance response; altered regional polyamine levels	Husain et al. 1994	Strengths: work uniquely covers effects of pyrethroids on different periods of perinatal development from shortly after conception to postweaning, and suggests that effects may depend on the exposure period (includes Malaviya et al. 1993). However, different compounds were utilized; effects on maternal parameters, general toxicity recorded; litter size adjusted to an average of 8 pups/litter
Rat (Charles Wistar)					Limitations: formulated products used; lack of relevant vehicle controls; general or less specific toxicity may be indicated by changes in fur development, pinna detachment; statistical models are often inappropriate; descriptions of comparisons (data sets) used for statistical tests are sometimes unclear or confusing; not clear that litter is the statistical unit
Fenvalerate	10 mg/kg. p.o.; corn oil	GD5–21 (gestational exposure) or PND1–15 (lactational exposure) Biochemical outcomes measured at 3 weeks of age	0 effect on dam weight, food/water intake, gestation length, no. of offspring, sex ratio Gestational exposure: ↓MAO, Na/K-ATPase activity; spiroperidol binding; ↑AChE activity Lactational exposure: ↓MAO, AChE activity; ↑spiroperidol, QNB binding	Malaviya et al. 1993	
Cypermethrin	15 mg/kg, p.o.; corn oil	GD5–21 (gestational exposure) or PND1–15 (lactational exposure) Biochemical outcomes measured at 3 weeks of age	0 effect on dam weight, food/water intake, gestation length, no. of offspring, sex ratio Gestational exposure: 0 effect on MAO, Na/K-ATPase, AChE activity; spiroperidol binding ↓ QNB binding Lactational exposure: ↓Na/K-ATPase, AChE activity; ↑ spiroperidol, QNB binding	Malaviya et al. 1993	
Rat (Wistar)					
*d*-Allethrin	0.43–74.2 mg/m^3^ Inhalation; unknown vehicle	PND10–16, 6hr/day	0 Effects on weight gain, motor activity, mAChR density when assessed on PND17 and 4 months	Tsuji et al. 2002	Strengths: measured air levels of allethrin during exposure; provides additional exposure information; multiple dose levels; litter controlled*^c^*
			0 effect in Morris water maze at 11 months		Limitations: absence of positive controls; this would demonstrate that lack of effect is true negative
Mouse (ICR)					
Permethrin (*cis* or *trans*)	Experiment 1: 0.33 to 33 μg/ml *cis*-permethrin or 33 μg/mL *trans*-permethrin in drinking water; 0.33 μg/mL DMSO vehicle	PND0–21	0 effect on weight in dam, pups; concentration-dependent decrease in c-*fos* mRNA in cerebellum at PND21; trend toward decrease in BDNF mRNA at PND21; 0 effect on β -actin mRNA	Imamura et al. 2002	Strengths: water consumption (ingested dose) measured; replication of c-*fos* decrease by different routes of exposure; similar findings following *in vitro* exposure to cerebellar granule cells (Imamura et al. 2000)
	Experiment 2: 1 mg/day *cis*-permethrin, p.o. corn oil	PND0–35	↓ c-*fos* mRNA at PND21 only; 0 effect on β-actin mRNA at any time		Limitations: did not use litter as statistical unit. 3–4 samples/litter; BDNF data variable
Mouse (NMRI)					
Deltamethrin	0.7 mg/ml p.o.; 20% fat emulsion (egg lecithin/peanut oil) Hypothermic, normothermic, and hyperthermic groups	PND10–16	Pup mortality in hypothermic groups (control and *S*-bioallethrin), including cannibalism; hypothermic pups displayed reduced motility; body weight gain PND10–17 was affected by conditions of hypothermia, hyperthermia; rectal temperature was affected by environmental temperature, differences in temperature between control and deltamethrin-treated animals were present in hypothermic but not hyperthermic animals; environmental temperature altered brain weight, with effects of *S*-bioallethrin and deltamethrin observed only in hypothermic animals; both deltamethrin and *S-*bioallethrin decreased brain/body weight ratios in hypothermic animals; QNB binding: on PND17, mAChR density was increased in both sexes by *S*-bioallethrin in hypothermic and normothermic groups; no differences were observed in the hyperthermic group or in the deltamethrin-treated groups	Pauluhn and Schmuck 2003	Strengths: technical compound of known purity used (99.8% for deltamethrin and 95.7% for *S*-bioallethrin); statistical analysis using ANOVAs; randomized selection of pups and dams for treatment groups from a pool. Limitations: pup mortality observed in control, *S-*bioallethrin groups with no information provided regarding number of pups lost/cannibalized; replacement pups came from a pool of pups that had been housed under “normal conditions,” which likely differed in temperature from group that lost pups (hypothermic pups); sample size for various end points is difficult to determine; examined only PND17 animals; not known if temperature differences could contribute to long-term changes in mAChR expression; randomized assignment of pups to dams does not control for maternal effects; did not demonstrate that typical p.o. dosing causes hypothermia; because of design of study (incomplete block design), comparisons between vehicle and pyrethroid treatments cannot be made; study design was to compare effects of different temperature conditions within these treatments
*S*-bioallethrin	0.7 mg/mL p.o.; 20% fat emulsion (egg lecithin/peanut oil) Hypothermic, normothermic and hyperthermic groups	PND10–16			

Abbreviations: 5HIAA, 5-hydroxyindoleacetic acid; 5HT, serotonin; AChE, acetylcholinesterase; ANOVA, analysis of variance; BBB, blood–brain barrier; BDNF, brain-derived neurotropic factor; *B*_max_, maximum number of binding sites; CTX, cortex; DA, dopamine; DMSO, dimethyl sulfoxide; EC, emulsifiable concentrate; GAP-43, growth-associated protein 43; GSH, glutathione; HP, hippocampus; HVA, homovanillic acid; i.p., intraperitoneal; LD_50_, dose lethal to 50%; LH, luteinizing hormone; MAO, monoamine oxidase; NA, noradrenaline; nAChR, nicotinic acetylcholine receptor; p.o., per os; STR, stratum.

a Publications by the same group of authors are indicated by shading; in some cases, comments are made on groups of papers published by the same group of authors rather than on individual papers.

b Not reported in original publication (Gupta et al. 1999a); data from A.K. Agarwal (personal communication).

c Not reported in original publication (Tsuji et al. 2002); data from R. Tsuji (personal communication).

**Table 4:** Summary of data from studies in NMRI mice (dosed once daily on PND10–16) submitted to the U.S. EPA.

Compound	Dose/route/vehicle	Effects	References	Comments
*d*-Allethrin	0.15, 4, or 100 mg/m^3^ 6 hr/day, inhalation; polyethylene glycol	PND17: motor activity: increased habituation in 0.15 mg/m^3^ females when compared to control; effects not dose-related; mAChR: 25% ↑in QNB in cortex, smaller changes in hippocampus and striatum; nAChR: 40–60% ↓in cortex, hippocampus, and striatum in both sexes; AChE: ↑ by 70–80% in striatum but not significant due to large variability; ChAT: 0 effect	Ivens et al., unpublished data	Strengths: technical compound, 95% purity; group sizes of 10; litter was statistical unit; good statistical analysis, males and females considered separately; second control group was included; closely replicates methodology of Eriksson and co-workers (see Table 2) for motor activity measurements
				Limitations: not peer-reviewed or published; some biochemical measurements were variable and not dose-related
		4 months: motor activity: no significant effects; mAChR: 0 effect; nAChR: large sporadic changes but no clear sex- or dose-related trends; AChE: 0 effect; ChAT: 0 effect		
Cyfluthrin	6, 15 or 50 mg/m^3^, 6 hr/day, inhalation; polyethylene glycol	All pups died in 50 mg/m^3^ dose group; 15 mg/m^3^ pups had clinical signs including “clonic seizures” (probably tremors and/or choreoathetosis); ↓pup weight in 15 mg/m^3^ and in 5 mg/m^3^ females	Jekat et al., unpublished data	Strengths: technical compound, 96.8% purity; group sizes of 10; litter was statistical unit; good within-lab replicability for motor activity [comparison of data with Ivens et al. (unpublished data)]; closely replicates methodology of Eriksson and co-workers (see Table 2) for motor activity measurements
		PND17: no measurements		Limitations: not peer-reviewed or published; only examined adults; general toxicity observed; QNB data variable, no dose-related effects, difficult to compare with other studies because presented either as dpm or percent of control
		4 months: motor activity: 15 mg/m^3^ females were hyperactive and had decreased habituation in horizontal and vertical v activity;mAChR: ↓QNB binding (not statistically significant) of ~22% in 15 mg/m^3^ males		

Abbreviations: AChE, acetylcholinesterase; ChAT, choline acetyltransferase; nAChR, nicotinic acetylcholine receptor.

**Table 5:** Summary of effects on mAChR and motor activity after developmental exposure to pyrethroids.

	MAChR expression*^a^*		Motor activity		
Compound*^b^*	Preweaning	Adult	Preweaning	Adult	Reference
*d*-Allethrin	↑CTX	0 CTX	↑HB	0	Ivens et al., unpublished data
*d*-Allethrin	0	0	ND	0	Tsuji et al. 2002
Bioallethrin	0 CTX	↑CTX*^c^*	ND	↑MA, ↓HB	Muhammad and Ray, unpublished data
Bioallethrin	ND	0 CTX	ND	↑MA, ↓HB	Talts et al. 1998
Bioallethrin/bioallethrin	ND	↑ CTX	ND	↑MA, ↓HB	
Bioallethrin	ND	↓CTX; 0 HP, STR	0 MA, 0 HB	↑MA, ↓HB	Eriksson and Fredriksson 1991
Bioallethrin	↑CTX	↓CTX	ND	↑MA, ↓HB	Ahlbom et al. 1994
Bioallethrin	↑CTX	ND	ND	ND	Eriksson and Nordberg 1990
*S*-Bioallethrin	↑CTX	ND	ND	ND	Pauluhn and Schmuck 2003
Cyfluthrin	ND	0 CTX	ND	In females,↑MA, ↓HB	Jekat et al., unpublished data
Cypermethin	↓STR (gestation experiment), ↑STR (lactation experiment)	ND	ND	ND	Malaviya et al. 1993
Deltamethrin	ND	↓HP	ND	ND	Aziz et al. 2001
Deltamethrin	↑CTX	↑CTX	ND	↓HB	Muhammad and Ray, unpublished data
Deltamethrin	ND	0 CTX, HP, STR	0 MA, 0 HB	↑MA, ↓HB	Eriksson and Fredriksson 1991
Deltamethrin	↓HP	ND	ND	ND	Eriksson and Nordberg 1990
Deltamethrin	0 CTX	ND	ND	ND	Pauluhn and Schmuck 2003
Fenvalerate	0 STR (gestation experiment), ↑STR (lactation experiment)	ND	ND	ND	Malaviya et al. 1993

Abbreviations: 0, end point was examined and was not affected by treatment; CTX, cortex; HB, habituation; HP, hippocampus; MA, motor activity; ND, not determined; STR, striatum.

a As measured by QNB binding.

b Compounds are arranged in alphabetical order.

c An increase in QNB binding was observed in one “cohort” but was not consistently observed in all “cohorts” in studies by this group.

See Table 3 for complete details.
